# Total Knee Arthroplasty in a Patient With Isolated Rheumatoid Arthritis of the Knee

**DOI:** 10.7759/cureus.83818

**Published:** 2025-05-09

**Authors:** Joshua L Dale, Zain Sayeed

**Affiliations:** 1 Osteopathic Medicine, William Carey University College of Osteopathic Medicine, Hattiesburg, USA; 2 Orthopedics, Doctors Hospital at Renaissance, Edinburg, USA

**Keywords:** msk radiology, ortho surgery, rheumatoid arthriitis, student education, total knee replacement (tkr)

## Abstract

Rheumatoid arthritis (RA) is a chronic autoimmune inflammatory disorder characterized by synovial inflammation, progressive bone and cartilage erosion, and eventual joint destruction. We present the case of a 59-year-old female with long-standing RA who was referred by her rheumatologist after developing advanced arthritis of the right knee. Despite appropriate medical management, including etanercept and methotrexate, the patient continued to experience significant pain and limited range of motion in the right knee. She subsequently underwent total knee arthroplasty (TKA), resulting in substantial improvement in both pain and mobility. Notably, this case is characterized by the isolated involvement of the right knee, despite the patient’s long-term adherence to her medication regimen. On physical examination, the patient did not demonstrate any reduction in the range of motion or deformity in other joints. This case highlights the importance of routine monitoring, imaging, and early intervention in patients with autoimmune inflammatory arthropathies, as well as the need for further research on surgical approaches for RA of the knee.

## Introduction

Rheumatoid arthritis (RA) is a chronic autoimmune inflammatory disorder characterized by persistent synovial inflammation and progressive erosion of bone and cartilage, ultimately leading to joint destruction [1]. Clinically, patients often present with morning stiffness, reduced joint mobility, fatigue, fever, and unintended weight loss [1]. Although RA is primarily diagnosed clinically, laboratory markers such as anti-cyclic citrullinated peptide (anti-CCP) antibodies and immunoglobulin M rheumatoid factor (RF) support the diagnosis, with approximately 75% of patients testing positive for these markers [2]. RA affects approximately 1% of the U.S. population, making it a relatively common condition encountered in clinical practice [1,2].

The 2010 American College of Rheumatology (ACR) and European League Against Rheumatism (EULAR) classification criteria assess RA severity based on joint distribution, serologic markers, symptom duration, and acute-phase reactants [2]. Joint involvement typically includes the metacarpophalangeal (MCP) and proximal interphalangeal (PIP) joints of the hands, sparing the distal interphalangeal (DIP) joints and wrists [2]. However, involvement of larger joints, such as the knees, is not uncommon. Studies have shown that up to 71% of patients with RA develop secondary osteoarthritis (OA) of the knee [3].

In advanced stages of RA, when conservative management fails, total knee arthroplasty (TKA) has proven to be an effective intervention for reducing pain and improving physical function [4]. However, performing TKA in RA patients presents unique challenges compared to the general population, including poor bone quality, increased risk of flexion contractures, patellar maltracking, and delayed healing due to long-term use of disease-modifying antirheumatic drugs (DMARDs) and corticosteroids [4].

In this case report, we present a patient with long-standing RA localized solely to the right knee referred to us by their rheumatologist, who experienced significant functional impairment and pain despite extensive conservative treatment. While RA often presents as a symmetric polyarthritis, this case is notable for isolated monoarticular knee involvement after two decades of disease. Given the severity of symptoms and limited mobility, the decision was made to proceed with the right TKA

## Case presentation

A 59-year-old Hispanic female presented to the clinic with complaints of right knee pain and limited mobility. She had previously been diagnosed with RA with an elevated rheumatoid factor and later tested positive for anti-CCP antibodies, confirming the diagnosis. In the past, her RA flares were managed with a combination of etanercept and methotrexate. Aside from RA, the patient had no other significant medical history. She denied smoking, alcohol use, or any prior trauma or falls. The patient reported progressively worsening right knee pain that did not improve despite undergoing six weeks of physical therapy as prescribed by her referring physician. She had also received two corticosteroid injections over a six-month period, which provided only minimal relief. At the time of presentation, her pain significantly interfered with daily activities and severely limited mobility.

On physical examination, the patient appeared healthy, with no other musculoskeletal abnormalities. Examination of the right knee revealed a limited range of motion in both flexion and extension, along with a varus deformity. Range of motion testing demonstrated flexion limited to 45 degrees and extension limited to 15 degrees. Tenderness was noted along both the medial and lateral joint lines. Ligamentous testing revealed no evidence of instability. Patient reported their pain routinely at 9/10 when weight bearing. As well, we tapped the knee and ordered analysis of the patient's synovial fluid. The lab results were consistent with inflammatory arthritis; results are summarized in Table 1.

**Table 1:** Analysis of right knee synovial fluid analysis.

Test	Result	Normal Reference Range
Appearance	Cloudy	Clear or slightly cloudy
Leukocyte Count	28,000 cells/µL	0-2,000 cells/µL
Neutrophils (%)	0.75	0-25%
Red Blood Cells (RBCs)	1,200 cells/µL	0-200 cells/µL
Glucose	60 mg/dL	50-100 mg/dL (slightly lower than serum)
Protein	5.2 g/dL	1.5-3.5 g/dL
Crystals	Negative	None (normal)
Gram Stain	No organisms	Negative (normal)
Culture	No growth after 48 hours	No growth (normal)
Complement C3	Decreased	90-180 mg/dL
Complement C4	Decreased	10-40 mg/dL

**Figure 1:**
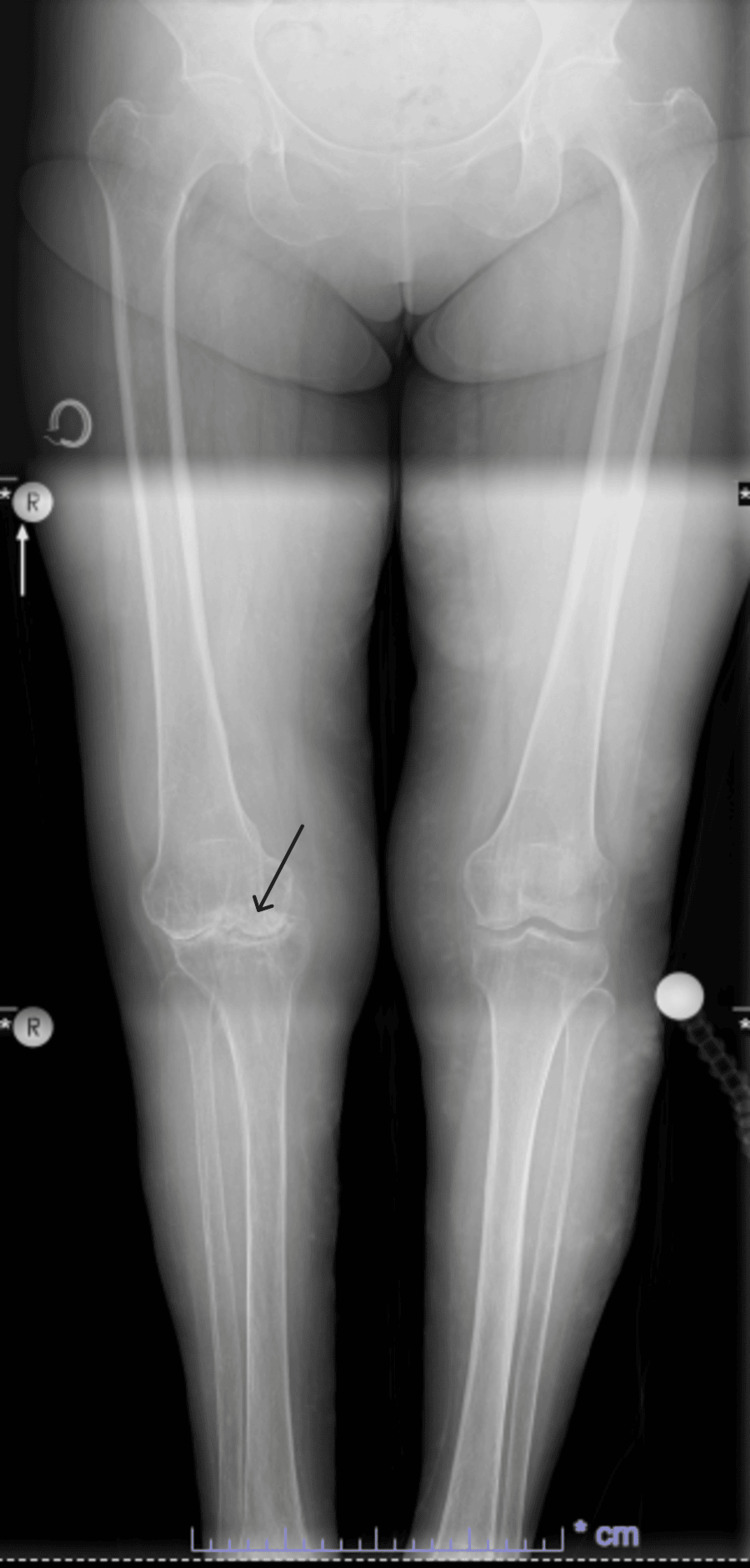
Plain radiography, AP, weight bearing view of bilateral knees. Presence of end stage arthritis of right knee.

**Figure 2:**
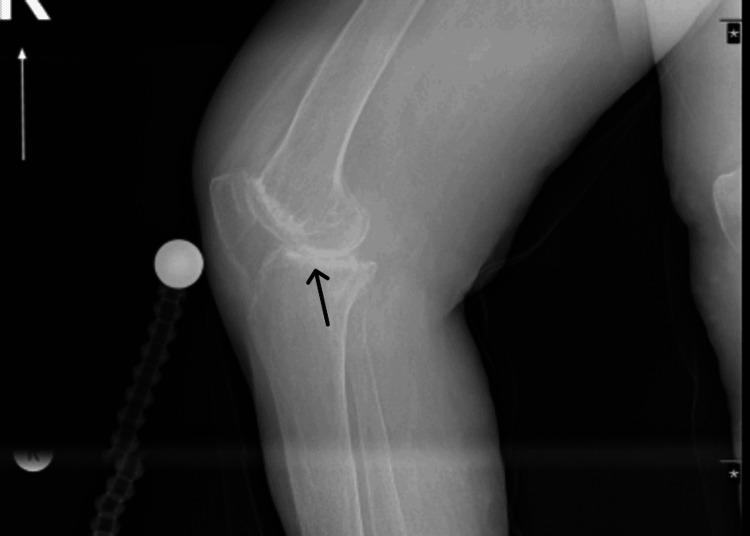
Plain radiography, lateral, weight bearing view, demonstrating bone on bone arthritis of the medial compartment.

**Figure 3:**
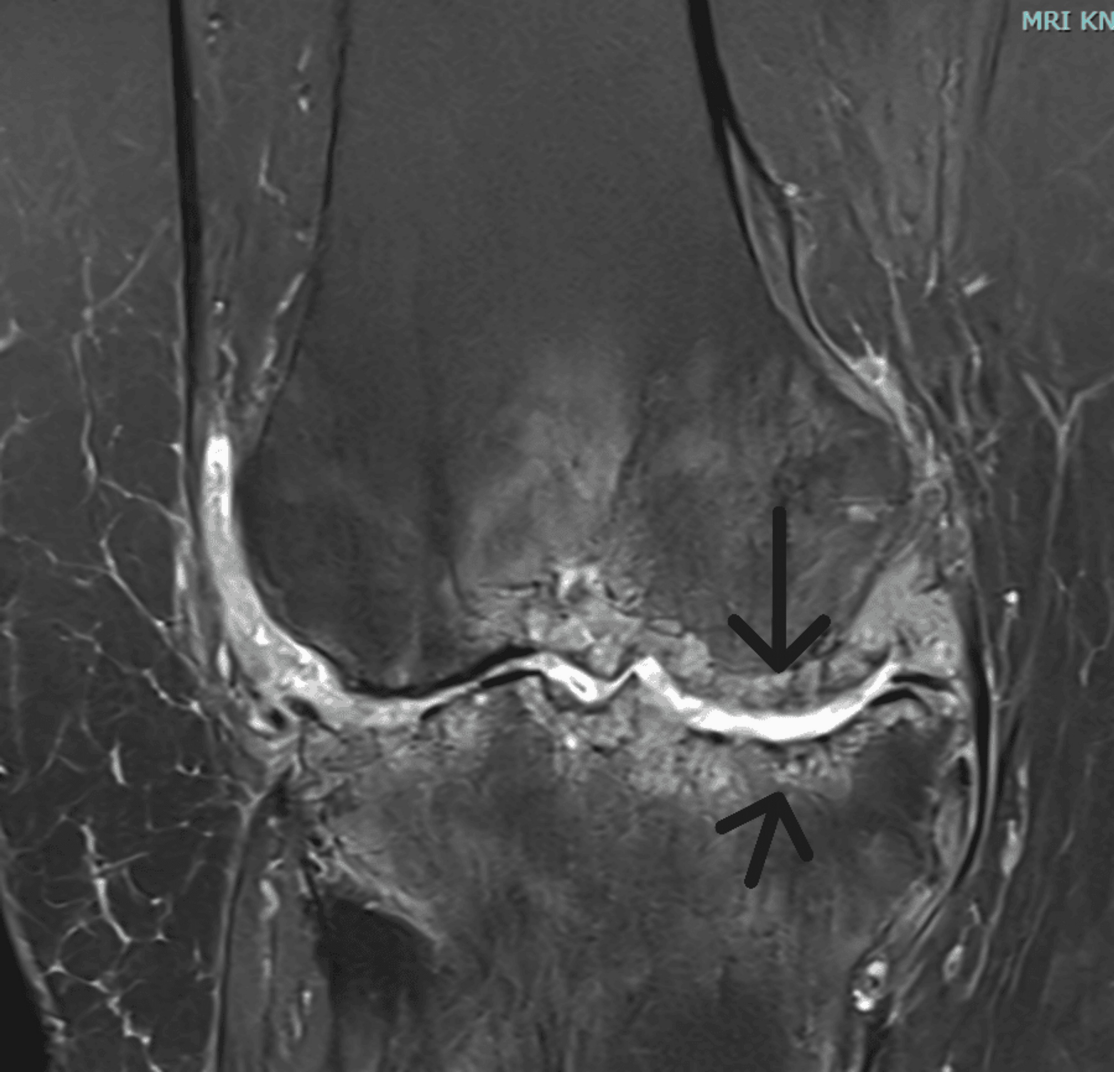
Τ2 weighted image, MRI of the right knee, coronal view. Arrows pointing towards evidence of joint line erosion.

**Figure 4:**
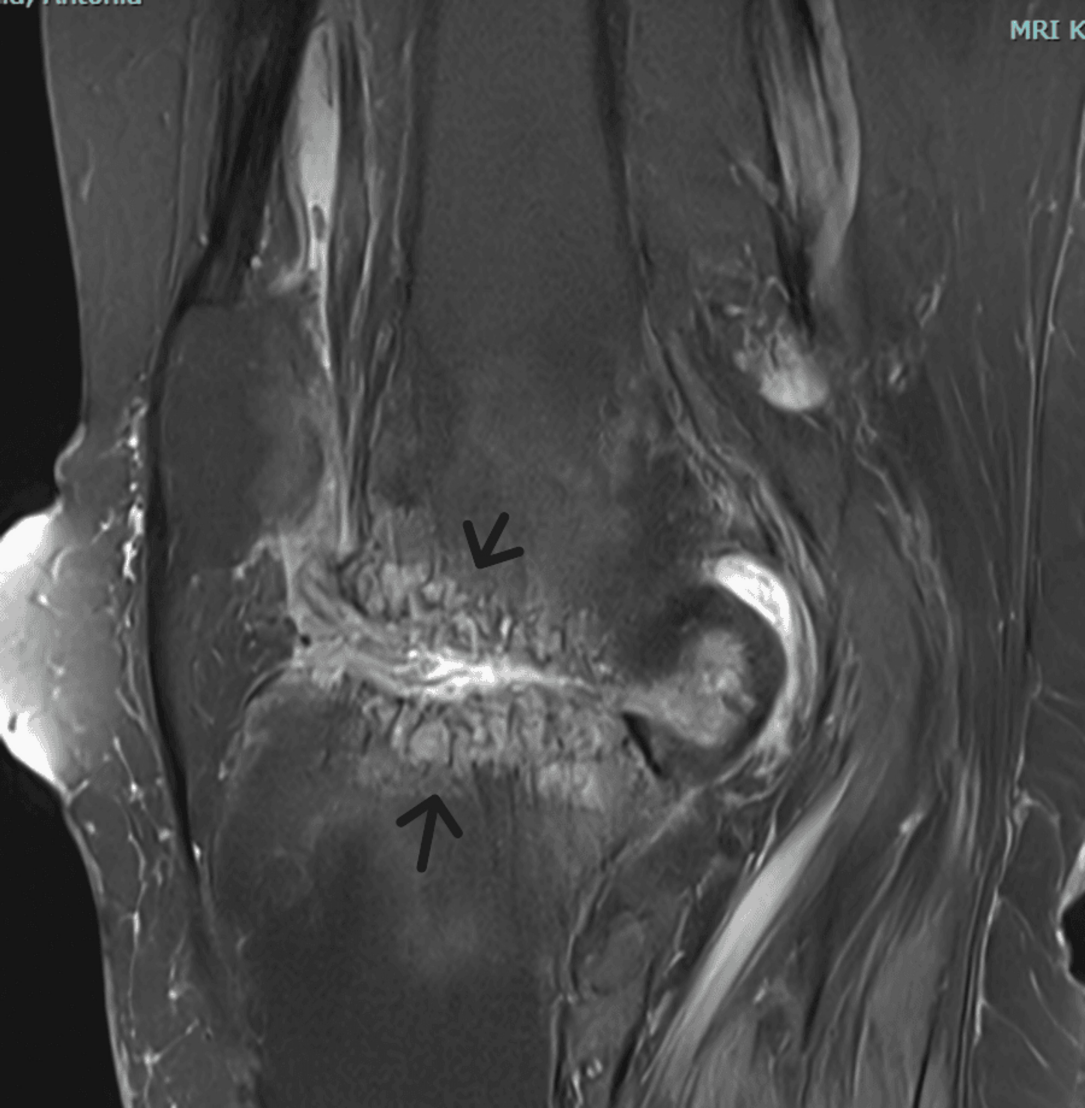
Τ2 weighted image. MRI of the right knee, sagittal view. Arrows pointing towards evidence of joint line erosion.

Radiographic imaging demonstrated medial joint space narrowing, a varus deformity of the right knee, and relative osteopenia (Figures 1, 2). MRI, which was ordered at the patient's request, revealed inflammatory and arthritic changes, predominantly affecting the medial compartment of the right knee (Figures 3, 4).

Based on clinical laboratory and imaging findings, a diagnosis of OA of the right knee was made. The patient was counseled on her condition and presented with several treatment options, including corticosteroid injection, additional physical therapy, or TKA. Following informed consent and a thorough discussion of risks and benefits, the patient elected to proceed with TKA. Despite medical advice to continue her RA medications perioperatively, the patient independently discontinued them four weeks before surgery, believing this would aid healing.

**Figure 5:**
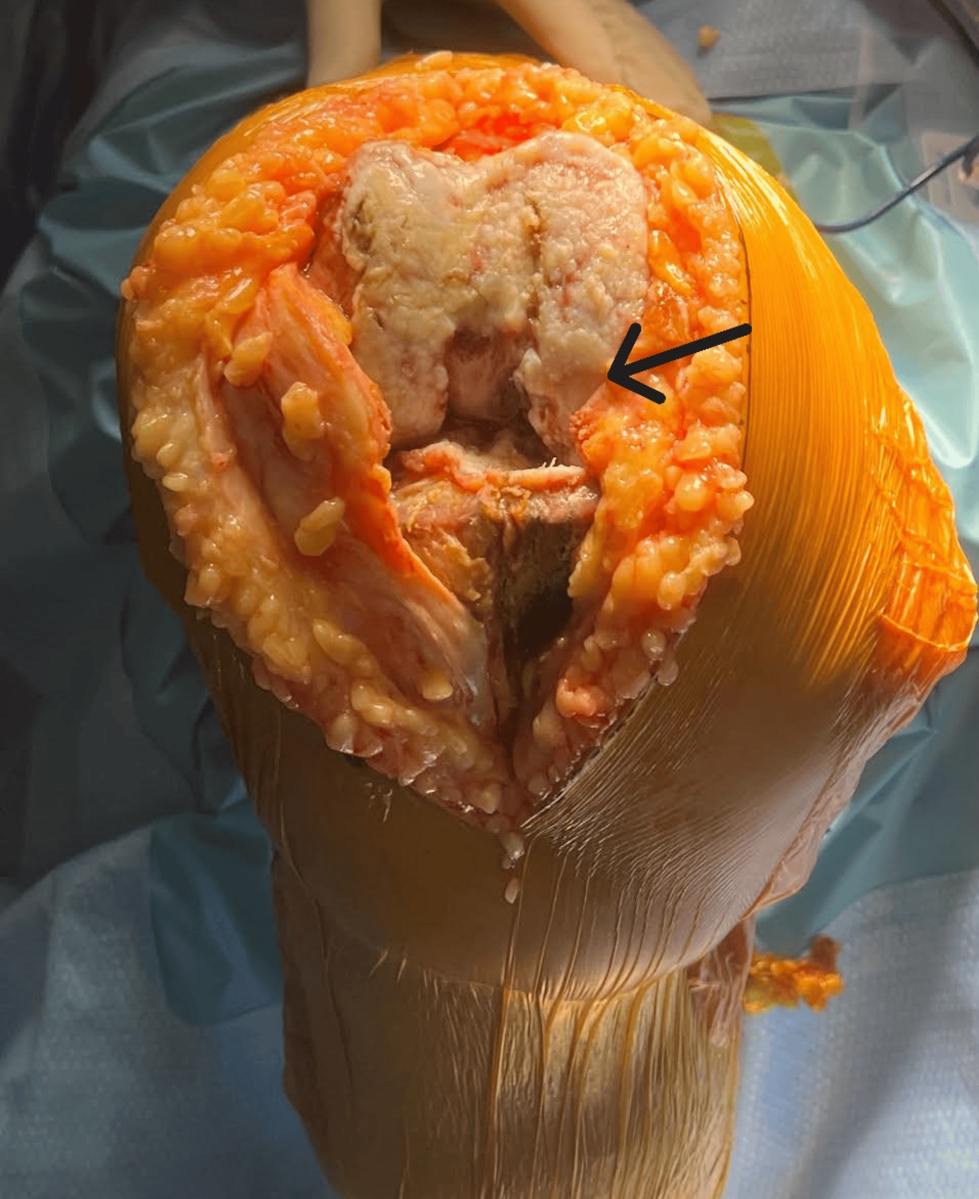
Intraoperative knee. Arrow pointing towards the medial joint line erosion.

**Figure 6:**
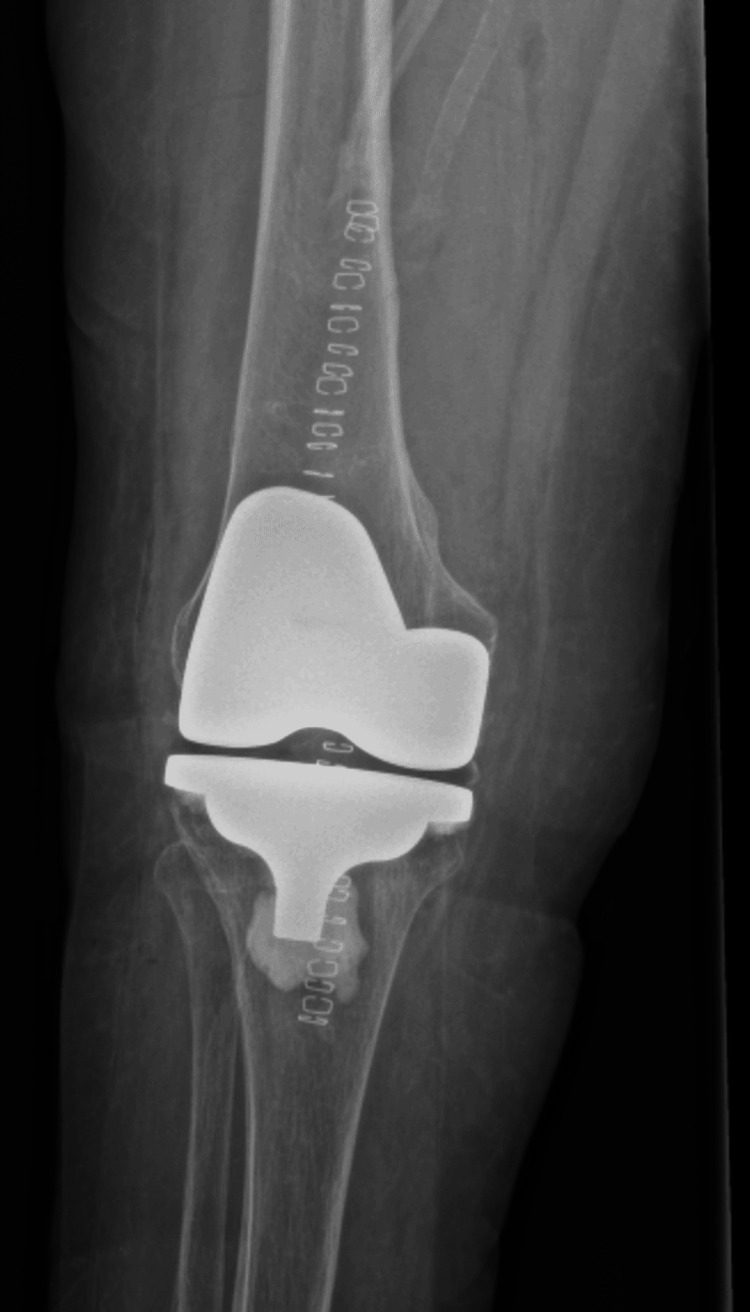
Postoperative plain radiography, AP, non-weight bearing view, demonstrating new prosthesis.

**Figure 7:**
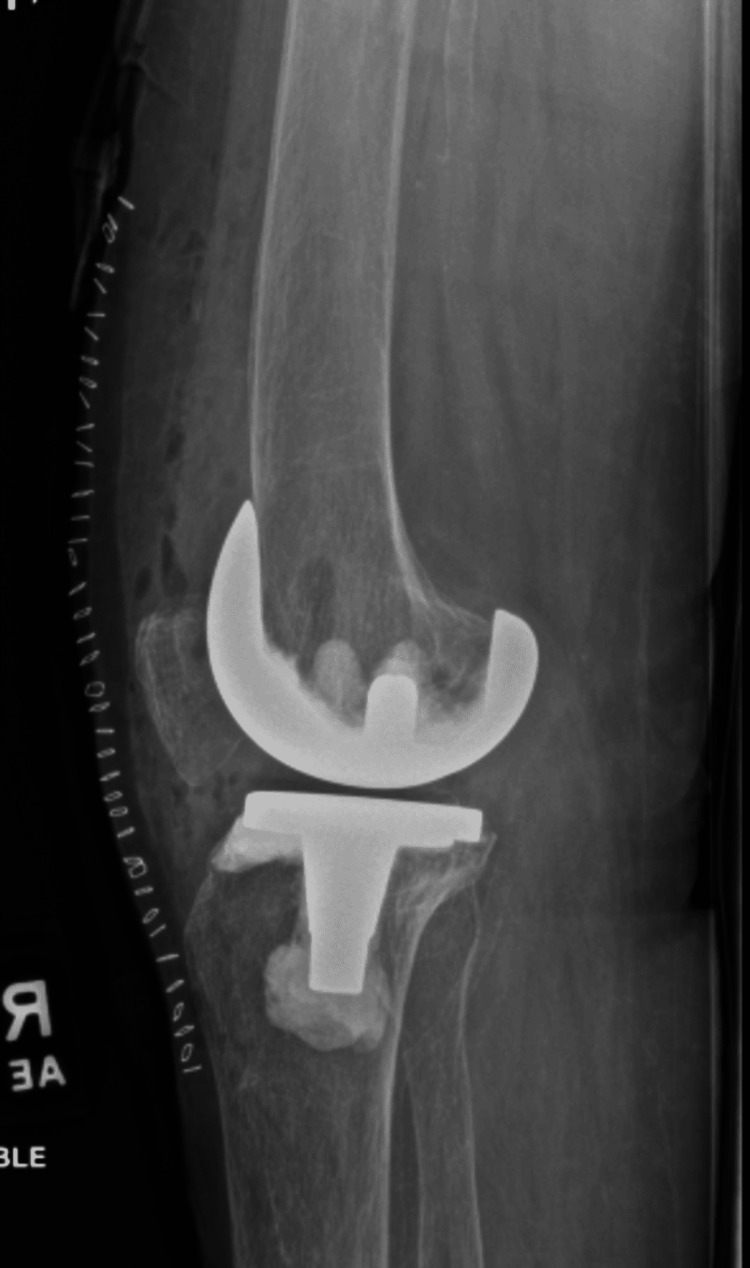
Postoperative plain radiography, lateral, non-weight bearing view, demonstrating new prosthesis.

The patient underwent a right TKA using a standard medial parapatellar approach. Upon further dissection, advanced arthritis and significant damage due to inflammation were noted in the right knee (Figure 5). During the procedure, it was observed that the patient's patella had not experienced the same level of wear as the rest of the knee, so it was decided to leave the patella intact. She was admitted postoperatively for 24 hours for observation, pain control, and physical therapy. The patient was counseled to begin her methotrexate the day after surgery and to resume her etanercept 14 days post op. The patient was discharged on postoperative day one with instructions to follow up in the clinic in 10 days. At her 10-day postoperative follow-up, she reported minimal pain and no longer required pain medications. Postoperative radiographs are shown in Figures 6, 7.

## Discussion

RA is a chronic, systemic inflammatory disease that often results in debilitating joint pain and deformity. More than half of patients with RA will undergo orthopedic surgery during their lifetime, with TKA among the most commonly performed procedures [5,6]. Our patient’s presentation was unusual in that she had serologically confirmed RA for over 20 years, yet only presented with deformity and symptoms in the right knee. Although rare, similar cases have been documented. Kim et al. reported a case of unilateral rheumatological changes in the left knee of a hemiplegic patient [7]. While our patient was fully ambulatory, this case similarly underscores the need for accurate diagnostics and individualized treatment planning. Long-term adherence to RA medications may have played a role in preventing more widespread joint involvement in our patient.

The diagnosis and classification of RA are guided by the 2010 ACR/EULAR Rheumatoid Arthritis Classification Criteria, which incorporate joint involvement, serologic markers, symptom duration, and acute-phase reactants [8]. These criteria are summarized in Table 2, with a score of ≥6 required for a definitive diagnosis. While the patient’s original diagnosis was based on serologic findings, her current presentation would yield a score of 5 under the ACR/EULAR classification, below the diagnostic threshold of 6. Although the patient's knee pathology was likely caused by RA, as indicated by the referral from her rheumatologist, we proceeded with a standard total knee approach, similar to that used in osteoarthritis.

**Table 2:** 2010 ACR/EULAR Rheumatoid Arthritis Classification Criteria RF = Rheumatoid Factor; ACPA = Anti-Citrullinated Protein Antibody; CRP = C-reactive Protein; ESR = Erythrocyte Sedimentation Rate [2,8]. Permission to use the table from Aletaha et al. [8] was obtained from Elsevier with the authors’ consent for inclusion in this submission.

Category	Criteria	Score
Joint Involvement	1 large joint	0
	2–10 large joints	1
	1–3 small joints (± large joint involvement)	2
	4–10 small joints	3
	>10 joints (including ≥1 small joint)	5
Serology	Negative RF and ACPA	0
	Low-positive RF or ACPA	2
	High-positive RF or ACPA	3
Symptom Duration	<6 weeks	0
	≥6 weeks	1
Acute Phase Reactants	Normal CRP and ESR	0
	Abnormal CRP or ESR	1

Challenges unique to RA patients

Patients with RA undergoing joint replacement face challenges not typically encountered in the general population, including higher risks of periprosthetic infection, prosthetic loosening, and poor bone quality due to chronic inflammation and corticosteroid use [9]. In our patient’s case, we opted to cement the prosthesis to mitigate the risk of prosthetic loosening and to compensate for poor bone stock. Recent studies suggest that the use of DMARDs, including biologics, does not significantly impair wound healing or increase the rate of surgical site infections [10]. Progressive joint damage in RA often results in structural deformities, particularly in weight-bearing joints. RA most commonly leads to a valgus deformity of the knee due to lateral compartment erosion and medial soft tissue attenuation [11]. This angular deformity can complicate both intraoperative management and postoperative recovery.

Surgical decision-making

We proceeded with TKA using the medial parapatellar approach based on the operating surgeon’s expertise and preference. This remains the most commonly used approach for TKA. However, Noguchi et al. demonstrated that the lateral approach may offer benefits in RA patients with valgus deformity, including better preservation of the medial collateral ligament (MCL) and medial patellofemoral ligament (MPFL) [11]. These structures are frequently compromised in RA patients, and additional surgical disruption could contribute to postoperative instability. Although our patient had a varus deformity rather than valgus, the decision-making process highlights the need to individualize surgical technique based on joint anatomy and RA progression.

## Conclusions

RA is a progressive autoimmune disease that often leads to joint destruction and functional impairment. TKA remains the definitive surgical intervention for patients with end-stage RA affecting the knee. While the medial parapatellar approach remains the standard, emerging evidence suggests that alternative approaches may offer advantages in managing specific RA-related deformities. Further research is needed to refine surgical strategies tailored to RA-associated deformities. This case also highlights the importance of imaging in patients with inflammatory arthropathies, particularly in those experiencing exacerbation of symptoms.
